# Safety of combined drug use in patients with cardiovascular and cerebrovascular diseases: an analysis based on the spontaneous reporting database of adverse drug reactions in Hubei Province

**DOI:** 10.3389/fphar.2024.1451713

**Published:** 2025-01-08

**Authors:** Jia Wang, Yuhang Zhao, Zherui Chen, Rui Huang

**Affiliations:** ^1^ Personnel section, Traditional Chinese and Western Medicine Hospital of Wuhan, Tongji Medical College, Huazhong University of Science and Technology, Wuhan, China; ^2^ School of Pharmacy, Tongji Medical College, Huazhong University of Science and Technology, Wuhan, China; ^3^ School of Statistics and Mathematics, Zhongnan University of Economics and Law, Wuhan, China

**Keywords:** cardiovascular diseases, cerebrovascular diseases, adverse drug reaction, association rule, Ω shrinkage measurement

## Abstract

**Objective:**

There is a lack of studies investigating the safety of combination regimens specifically for cardiovascular and cerebrovascular diseases. This study aimed to evaluate the safety of combination drugs for cardiovascular and cerebrovascular diseases using real-world data.

**Methods:**

We analyzed adverse drug reaction data received by the Hubei Adverse Drug Reaction Center from the first quarter of 2014 to the fourth quarter of 2022. The safety of combined drugs for cardiovascular and cerebrovascular diseases in different people was assessed using the association rule method and Ω shrinkage measurement.

**Results:**

A total of 53,038 reports were included in this study, revealing 9 signals of adverse reactions caused by combination drugs. The strongest signal found in this study was jaundice caused by the combination of amlodipine and atorvastatin (Ω 0.025:3.08, lift: 1116.69, conviction: 1.75). Additionally, the combination of aspirin with other drugs was associated with hemorrhaging in various organs. Female patients showed a cold signal when taking vitamin C and vitamin B6 together compared to male patients (Ω 0.025:0.89, lift: 7.15, conviction: 1.12). Patients under 60 years old had a palpitations signal when combining eritrea bei sha Tanzania and felodipine (Ω 0.025:0.41, lift: 14.65, conviction: 3.8), and an erythema signal when combining nifedipine (Ω 0.025:0.23, lift: 8.17, conviction: 1.077).

**Conclusion:**

Among the 9 signals identified in this study, 4 were off-label adverse drug reactions that require further clinical research for exploration and confirmation, in order to provide more scientifically informed drug labeling. Five adverse events associated with aspirin-induced bleeding were identified. Notably, different adverse drug reactions were observed in different populations, suggesting the need for future studies to expedite the development of personalized medicine.

## 1 Introduction

Cardiovascular and cerebrovascular diseases, as non-communicable diseases, significantly impact global health (Aday and Matsushita, 2021; Clark, 2013; Sacco et al., 2016; Wang et al., 2021),They are the primary contributors to mortality and morbidity in developed and developing nations (Virani et al., 2020; Zhao et al., 2019). A global study spanning 204 countries revealed a significant rise in the prevalence of cardiovascular diseases from 1990 to 2019, with cases increasing from 271 million to 523 million. Concurrently, the number of deaths surged from 12.1 million to 18.6 million (Roth et al., 2020). WHO reports that approximately 17.9 million people died from cardiovascular disease in 2019, accounting for 32% of all deaths worldwide (World Health Organization, 2021). From 1990 to 2019, the number of deaths caused by cardiovascular diseases in China witnessed a significant increase, rising from 2.42 million to 4.58 million (Institute for Health Metrics and Evaluation, 2019).

Cardiovascular and cerebrovascular diseases make a substantial contribution to the increasing healthcare costs. In the United States, Cardiovascular disease is the leading cause of death, followed by stroke as the fifth leading cause, together accounting for around one-third of all annual deaths (Calitz et al., 2021). Cerebrovascular disease treatment in the European Union will cost $282 billion annually (Luengo-Fernandez et al., 2023), indicating the substantial health and economic burdens posed by cardiovascular and cerebrovascular diseases worldwide.

Cardiovascular and cerebrovascular diseases predominantly manifest as chronic conditions, with drug therapy serving as the primary mode of treatment (Li. et al., 2022). Patients diagnosed with these diseases often rely on combination drug therapies to manage their multiple conditions and enhance treatment effectiveness (Gallagher et al., 2020; Millenaar et al., 2021; Nussbaumer et al., 2016; Villen et al., 2022; von Lueder and Atar, 2014; Wiggins et al., 2016). However, during the initial stages of drug development, the evaluation process primarily focuses on individual drugs, while the assessment of combined drug therapies remains limited. The utilization of combination drug therapies has been associated with an increased risk of adverse drug reactions (Davies and O'Mahony, 2015; Dow et al., 2023; Gutiérrez-Valencia et al., 2018; Koehl, 2023; Osanlou et al., 2022; Verde et al., 2019; Zhao et al., 2023). Such reactions are common in patients undergoing combination therapy, and safety concerns have specifically been raised in relation to cerebrovascular disease patients (Gallagher et al., 2020; Krishnaswami et al., 2019; Millenaar et al., 2021).

Consequently, there exists a lack of understanding regarding the changes in pharmacokinetics, efficacy, and toxicology associated with combined drug therapies, as well as their safety implications. This knowledge gap not only hinders the alleviation of patients’ symptoms but also introduces additional complications caused by adverse drug interactions (Ozokcu et al., 2024; Stollberger et al., 2023; von Lueder and Atar, 2014; Wiggins et al., 2016).

Currently, safety testing of drugs primarily occurs prior to their availability. However, the existing safety testing procedures suffer from two notable drawbacks. Firstly, safety and efficacy tests predominantly take place during clinical trials, which are conducted under ideal clinical conditions and may not accurately reflect real-world scenarios. Secondly, current safety testing practices primarily focus on assessing the safety of individual drugs, with limited emphasis on evaluating the safety of combined drug therapies. Consequently, there is a pressing need to enhance safety research pertaining to diseases that frequently require the administration of multiple drugs.

Several signal mining methods are employed in the analysis of drug combinations, encompassing techniques such as baseline modeling, interactive signal segmentation, logistic regression, Ω shrinkage measurement, and association rules, among others.

The Ω shrinkage measurement, endorsed by the WHO Uppsala Testing Center for Drug-Drug Interactions (DDI) signal analysis, is regarded as a conservative approach to signal detection (Noguchi et al., 2019; Noguchi et al., 2020; Noren et al., 2008). The association rules, differs from traditional signal mining algorithms in that it does not require the creation of combinations for every instance of “adverse drug reaction” within a large database. As a result, it addresses the limitations of traditional signal mining algorithms that fail to identify potential adverse reaction signals at an early stage. The process of association rule mining can be divided into two steps. Firstly, frequent item sets are identified, followed by the generation of association rules based on these frequent item sets. Subsequently, association rules are filtered using specified indicators.

Heba Ibrahim proposed a hybrid Apriori algorithm that effectively reduces computational costs while maintaining an average signal detection capability of 85% accuracy, 81% sensitivity, and 84% recall (Ibrahim et al., 2016). Wang Chao conducted simulations using Monte Carlo to generate drug Adverse Drug Event (ADE) reports and found that association rules demonstrated optimal comprehensive signal detection ability (Wang et al., 2012). In a study by Sindhu et al., association rules were employed to detect Drug-Drug Interaction (DDI) signals in the first three quarters of the FAERS database in the United States in 2012. They successfully detected 70% of DDI signals, outperforming traditional ROR\MHRA and BCPNN algorithms in terms of sensitivity, specificity, and overall effectiveness in signal detection of Adverse Drug Reactions (ADR) (Sindhu and Kannan, 2013). In the present study, both Ω contraction measurement and association rules were utilized for detecting adverse reaction signals associated with combined drug usage.

## 2 Materials and methods

### 2.1 Data source and preprocessing

The data used in this study were obtained from the Adverse Drug Reaction Monitoring Center of Hubei Province, covering the period from January 2014 to December 2022. These reports were collected from various sources, including medical institutions, enterprises, and residents. It is worth noting that 99% of the reports were provided by medical professionals, which enhances the credibility of the reported relationship between drugs and adverse events. Each report includes essential patient information, such as the patient’s basic details, disease type, specific drug involved, adverse reaction experienced, and the evaluated relationship between the drug and the adverse reaction. These relationships are categorized as certain, probable, possible, unlikely, or pending confirmation.

Inclusion criteria: 1) Reports entered into the system between the years 2014 and 2022. 2) Reports pertaining to patients diagnosed with cardiovascular and cerebrovascular diseases. 3) Reports that evaluate the relationship between drugs and adverse reactions as certain, probable, or possible.

Exclusion criteria: 1) Reports entered into the system before 2014 or after 2022; 2) Reports unrelated to cardiovascular and cerebrovascular diseases. 3) Reports where the relationship between drugs and adverse reactions is assessed as unlikely or pending confirmation.4) Reports where drug information, adverse reaction information, basic information missing.

The data was thoroughly cleaned and preprocessed prior to analysis to ensure its usability. In order to ensure uniformity, drug names in the reports were standardized using the National Medical Products Administration Data Query System. Furthermore, adverse drug reactions (ADRs) and their corresponding clinical manifestations were standardized for consistency using the Medical Dictionary for Regulatory Activities (MedDRA).

### 2.2 Data analysis

A descriptive analysis was conducted on multiple variables, including patient gender, age, medication usage, and information pertaining to adverse reactions found in the reports. To identify signals within combined medication usage in patients with cardiovascular and cerebrovascular diseases, both association rules and the Ω shrinkage measure method were employed to mine the signal of the full data, and the presence of signals served as the criterion for both mining methods in this study.

#### 2.2.1 Association rules

The main objective of this study was to examine the occurrence of adverse events (AE) resulting from the combined usage of drug 1 and drug 2. To accomplish this, association rules in the form of drug 1∩drug 2→AE were utilized. The criteria employed for detecting association rules in this study were “lift” and “conviction”. These measures assist in assessing the strength of the association between the drug combination and the incidence of adverse events.

Equations 1–4 present the standard measurement metrics for association rules along with their corresponding calculation formulas (Noguchi et al., 2019; Yamamoto et al., 2023).

The support represents the frequency at which the rule appears in the dataset. T represents the total number of item sets in the dataset:

$$\text{Support}\left(\text{drug}1\&\text{drug}2\to \text{AE}\right)=\frac{drug1\&drug2\to AE}{T}$$ (1)

Confidence represents the conditional probability of event B occurring given condition A:

$$\text{Confidence}\left(\text{drug}1\&\text{drug}2\to \text{AE}\right)=\frac{Support\left(drug1\&drug2\to AE\right)}{Support\left(drug1\&drug2\right)}$$ (2)

Lift represents the relative increase in the probability of event B occurring when condition A is present compared to when it is absent:

$$\text{Lift}\left(\text{drug}1\&\text{drug}2\to \text{AE}\right)=\frac{confidence\left(drug1\&drug2\to AE\right)}{support\left(AE\right)}$$ (3)

Conviction represents the ratio of the probability of the occurrence of condition A given event B to the probability of event B occurring independently:

$$\text{Conviction}\left(\text{drug}1\&\text{drug}2\to \text{AE}\right)=\frac{1-Support\left(AE\right)}{1-confidence\left(drug1\&drug2\to AE\right)}$$ (4)

This study utilized “Lift >1,” “Conviction >1,” and “N ≥ 3″as the criteria for detecting association rules.

#### 2.2.2 Ω shrinkage measure

The signal value was calculated by tabulating and summarizing the frequency of each case report, as shown in Table 1.

**TABLE 1:** Four-by-two contingency table for the evaluation of drug-drug interaction.

	Target AE	All other AEs	Total
Drug 1 and Drug 2	n_111_	n_110_	n_11+_
Only drug 1	n_101_	n_100_	n_10+_
Only drug 2	n_011_	n_010_	n_01+_
Neither Drug 1 or Drug 2	n_001_	n_000_	n_00+_
Total	n_++1_	n_++0_	n_+++_

AE: adverse event; n: the number of reports(e.g. n_111_: the number of drug 1 and drug 2 induced target AE reports, n_110_:the nmber of other AE induced by drug 1 and drug 1).

The Ω shrinkage measure was calculated using the observed 4 × 2 contingency table (Table 1) to assess the individual or combined impacts of drugs. The specific procedure for computing the signal can be found in Equations 5, 6 (Noguchi et al., 2019).

$$\Omega =\log2\frac{n111+0.5}{E111+0.5}$$ (5)

n111: the number of drug D1 and drug D2 induced target AE reports.E111:the expected number of drug D1 and drug D2 induced target AE reports

$$\Omega 0.025=\Omega -\frac{\varphi \left(0.975\right)}{\log\left(2\right)\sqrt{n_{111}}}$$ (6)

where φ(0.975) represents the 97.5% of the standard normal distribution,and Ω0.025 > 0 is utilized as the threshold for detecting signals of concomitant use with drug D1 and drug D2.

#### 2.2.3 Stratified analysis

We performed signal mining independently for cardiovascular and cerebrovascular patients across various gender and age groups to investigate the variability in the occurrence of adverse drug reactions among different populations.

The presence of signals served as the criterion for both mining methods in this study. All data analyses were donducted using R software version 4.0.2.

## 3 Result

### 3.1 Basic information on spontaneous reporting

In this study, a total of 53,038 adverse reaction reports were selected from a pool of 810,814 reports, resulting in 78,167 cases of drug-adverse reaction combinations, Table 2 shows some example reports in the cardiovascular and cerebrovascular diseases case table. Excluding the 59 cases of patients with unknown gender, it was found that the number of female patients who experienced ADRs (27,717) was slightly higher than that of male patients (25,262), with a male-to-female ratio of 1.10:1, indicating a small difference. Among the remaining reports, excluding one with unknown age, there were 36,309 adverse reaction reports from individuals aged 60 and above, and 16,729 adverse reaction reports from individuals aged below 60.

**TABLE 2:** Some reports in the cardiovascular and cerebrovascular diseases case table.

ID	Reporting year	Age	Sex	Reporting unit category	Disease	Drugs	ADES
***202200032	2022	77	F	Hospital	Cerebral Infarction, Coronary Heart Disease	Xuesaitong For Injection, Sodium Chloride Injection	Local Redness
***202100006	2021	48	M	Pharmaceutical Enterprise	Hypertension, Hyperlipemia	Amlodipine, Atorvastatin	Jaundice
***202100007	2021	55	F	Hospital	Hypertension	Amlodipine	Rash
***202000033	2020	59	M	Hospital	Acute Myocardial Infarction	Aspirin, Ticagrelor	Lower Gastrointestinal Bleeding (General)
***201800235	2018	87	F	Pharmaceutical Enterprise	Arrhythmia, Lung Infection, Hyponatremia,Hypoproteinemia,Basilar- Hemorrhage	Potassium Chloride Injection, Potassium Magnesium Aspartate Injection, Inosine Injection, Sodium Chloride Injection, Vitamin C Injection	Shiver, Tachypnea

Among the 78,167 drug-adverse reaction combinations, there were 70,642 cases (90.3%) where one drug was combined with an adverse reaction, 6,134 cases (7.8%) where two drugs were combined with an adverse reaction, and 1,391 cases (1.8%) where three or more drugs were combined with an adverse reaction. Nifedipine appeared most frequently, with a total of 7,397 occurrences, followed by isosorbide mononitrate (3,259 times), Xiangdan (2,393 times), and aspirin (1,986 times). Table 3 displays the top 10 drugs.

**TABLE 3:** Drugs of the reports.

Drug	Frequency	Perecentage
Nifedipine	7397	9.44%
Isosorbide mononitrate	3259	4.16%
Xiangdan	2393	3.05%
Aspirin	1986	2.54%
Shenmai	1964	2.51%
Ginkgo Leaf Extract and Dipyridamole	1618	2.07%
Levofloxacin	1608	2.05%
Xuesaitong	1406	1.79%
Lodixanol	1335	1.70%
Salvia miltiorrhiza	1223	1.56%

The adverse drug reactions (ADRs) and their clinical manifestations were standardized using the Medical Dictionary for Regulatory Activities (MedDRA). The results indicate that pruritus was the most frequent adverse reaction, occurring 6,161 times, followed by headache (5,732 times), rash (5,062 times), and nausea (4,584 times). A total of 26 categories of system organ damage were identified, predominantly including skin and subcutaneous tissue diseases (19.39%), gastrointestinal system diseases (17.96%), various neurological system diseases (15.16%), and systemic diseases and various reactions at the administration site (14.55%), among others. Table 4 presents the top 10 major adverse reactions along with their corresponding types of system organ damage.

**TABLE 4:** Frequently reported ADRs (TOP10).

ADR	Frequency	Perecentage	SOC
Pruritus	6161	7.88%	Skin and subcutaneous tissue disorders
headache	5732	7.33%	Nervous system disorders
rash	5062	6.48%	Skin and subcutaneous tissue disorders
nausea	4584	5.86%	Gastrointestinal disorders
dizziness	4226	5.41%	Cardiac disorders
hypersensitivity	4109	5.26%	Immune system disorders
Palpitations	4076	5.21%	Cardiac disorders
chest discomfort	3090	3.95%	Cardiac disorders
Flushing	2783	3.56%	General disorders and administration siteconditions
Vomiting	2768	3.54%	Gastrointestinal disorders

A total of 7,525 combinations of drug-ADRs were identified in the study. The combinations with the highest frequency were Salvia miltiorrhiza and Captopril with cough (27 times), followed by Salvia miltiorrhiza and Nifedipine with flushing (19 times), and Salvia miltiorrhiza and Nifedipine with headache (17 times). Table 5 presents the top 10 combinations of drug-adverse reaction interactions.

**TABLE 5:** Frequency reported drug-adverse reaction interactions.

Combination of drugs	ADRs	Frequency
Salvia miltiorrhiza and captopril	Cough	27
Salvia miltiorrhiza and Nifedipine	Flushing	19
Salvia miltiorrhiza and Nifedipine	headache	17
Aspirin and Clopidogrel	Gastrointestinal haemorrhage	16
Amlodipine and Atorvastatin	Jaundice	13
Aspirin and Clopidogrel	Upper gastrointestinal haemorrhage	12
Isosorbide mononitrate and Nicorandil	Headache	12
Aspirin and Ticagrelor	Gastrointestinal haemorrhage	12
Aspirin and Ticagrelor	Epistaxis	11
captopril and Nifedipine	Cough	11

### 3.2 Signal mining results of cardiovascular and cerebrovascular patients

A total of 54 signals were discovered using the Ω shrinkage measure model. A higher Ω_0.025_ value indicates a stronger association between the drug combination and ADR. Through association rule signal mining and after removing redundant rules, a total of 39 signals were discovered. In this study, lift was used to determine the strength of the association signals, where a higher lift value indicates a stronger association. A total of 9 signals were found by association rules and omega contraction measurements at the same time, and amlodipine combined with atorvastatin caused jaundice in both models was the strongest signal; secondly, aspirin combined with ticagrelor caused epistaxis in both models also showed strong signals, and the rest of the combined signal intensity ranked differently in the two models, as shown in Table 6 for details.

**TABLE 6:** Signals caused by concomitant drug use.

Combination of drugs	ADRs	n111	Ω_0.025_	Lift	Conviction	Confidence
Amlodipine and Atorvastatin	Jaundice	12	3.08	1116.69	1.75	0.43
Aspirin and Ticagrelor	Epistaxis	11	1.04	89.14	1.09	0.09
Aspirin and Clopidogrel	Petechiae	4	0.14	63.81	1.05	0.05
Aspirin and Clopidogrel	Haematochezia	13	0.04	55.83	1.06	0.06
Aspirin and Ticagrelor	Haemorrhage	10	0.04	45.31	1.07	0.07
Aspirin and Clopidogrel	Haemoptysis	6	0.36	44.01	1.033	0.03
Aspirin and Ticagrelor	Gingival bleeding	8	0.02	36.78	1.08	0.08
Nifedipine and Aspirin	Skin reaction	6	0.18	11.60	1.11	0.11
Vitamin B6 and Vitamin C	Chills	6	1.02	7.49	1.13	00.13

### 3.3 Signal values by patient gender

Among male patients, a total of 7 signals were discovered, and the strongest signal combination remains to be Amlodipine and Atorvastatin with jaundice (Ω0.025: 2.55, lift: 908.32, conviction: 2.00). The next strongest signals were various bleeding events caused by the combination of aspirin with Ticagrelor or Clopidogrel at different sites. Table 7 presents the relevant details.

**TABLE 7:** Signals of caused by concomitant use in males.

Combination of drugs	ADRs	n111	Ω_0.025_	Lift	Conviction	Confidence
Amlodipine and Atorvastatin	Jaundice	8	2.55	908.32	2.00	0.5
Aspirin and Ticagrelor	Cerebral haemorrhage	3	0.74	205.56	1.04	0.04
Aspirin and Ticagrelor	Epistaxis	8	0.455	73.41	1.11	0.10
Aspirin and Ticagrelor	Petechial rash	3	0.05	67.45	1.03	0.03
Aspirin and Clopidogrel	Melaena	7	0.01	55.97	1.05	0.05
Aspirin and Ticagrelor	Gastrointestinal haemorrhage	12	0.15	44.54	1.14	00.13
Aspirin and Ticagrelor	Haemoptysis	5	0.39	39.97	1.05	0.005

Among female patients, a total of 5 signals were discovered, and the most common signal remains to be Amlodipine-Atorvastatin and jaundice (Ω0.025: 1.46, lift: 1391.53, conviction: 1.50). In addition to the identified bleeding signals caused by the combination of aspirin with other drugs, there is also a signal of chills caused by the combination of vitamin B6 and vitamin C, as detailed in Table 8.

**TABLE 8:** Signals of caused by concomitant use in women.

Combination of drugs	ADRs	n111	Ω_0.025_	Lift	Conviction	Confidence
Amlodipine and Atorvastatin	Jaundice	4	1.46	1391.53	1.50	0.33
Aspirin and Ticagrelor	Upper gastrointestinal haemorrhage	5	0.08	121.71	1.11	0.10
Aspirin and Ticagrelor	Haemorrhage	6	0.66	102.24	1.14	0.12
Aspirin and Clopidogrel	Upper gastrointestinal haemorrhage	7	0.18	82.67	1.07	0.07
Vitamin B6 and vitamin C	Chills	5	0.89	7.15	1.12	0.12

### 3.4 Signal values by patient age

Among patients aged 60 and above, a total of 3 signals were discovered. These include Amlodipine-Atorvastatin and jaundice (Ω0.025: 1.80, lift: 1061.53, conviction: 1.56), Aspirin-Ticagrelor and nosebleeding, as well as the previously identified signal in female patients, Vitamin B6-Vitamin C and chills (Ω0.025: 0.27, lift: 7.99, conviction: 1.17). The details are shown in Table 9.

**TABLE 9:** Signals of caused by concomitant use in people over 60 years old.

Combination of drugs	ADRs	n111	Ω_0.025_	Lift	Conviction	Confidence
Amlodipine and Atorvastatin	Jaundice	5	1.80	1061.53	1.56	0.36
Aspirin and Ticagrelor	Epistaxis	8	1.09	95.63	1.10	0.09
Vitamin B6 and vitamin C	Chills	5	0.27	7.99	1.17	0.17

Among patients aged below 60, a total of 4 signals were discovered. Two newly discovered signals include the combination of Irbesartan-Amlodipine and palpitations, as well as the combination of Salvia miltiorrhiza-Nifedipine and erythema. Please refer to Table 10 for more details.

**TABLE 10:** Signals of caused by concomitant use in people below 60 years old.

Combination of drugs	ADRs	n111	Ω_0.025_	Lift	Conviction	Confidence
Amlodipine and Atorvastatin	Jaundice	7	2.35	1024.08	2.00	0.50
Aspirin and Clopidogrel	Haematochezia	4	0.80	121.37	1.10	0.09
Irbesartan and Felodipine	Palpitations	3	0.41	14.65	3.80	0.75
compound Danshen tablets and Nifedipine	Erythema	5	0.23	8.17	1.077	00.08

## 4 Discussion

In this study, signal mining was conducted on the entire population as well as subgroups including males, females, individuals under 60 years old, and individuals over 60 years old. The number of signals obtained was relatively small. This can be attributed to the study’s requirement that signals must satisfy both association rules and the criteria of Ω shrinkage measurement. It is worth noting that Ω shrinkage measurement was considered a more conservative signal mining method.

The strongest signal observed in this study was the occurrence of jaundice when amlodipine was combined with atorvastatin. No adverse reactions were found in Micromedex (Merative. 2024) and other drug websites. Considering the pharmacokinetic and pharmacodynamic properties of amlodipine and atorvastatin, these two drugs are commonly used for the management of cardiovascular disease. Relevant studies have demonstrated their effectiveness in controlling blood pressure and lowering low-density lipoprotein-C levels in patients with concomitant hypertension and dyslipidemia (Ali et al., 2023; Jin et al., 2020; Lin et al., 2024; Lindgren et al., 2009; Richard Hobbs et al., 2009). Moreover, previous studies have reported the safety of combined treatment with these two drugs, with adverse reactions including liver enzyme abnormalities, respiratory infections, peripheral edema, headaches, myalgia, accidental injuries, dizziness, diarrhea, nausea, and skin rashes (Blank et al., 2005; Flack et al., 2008; Jung et al., 2023; Lee et al., 2011) Although few studies have reported jaundice specifically associated with the combination of amlodipine and atorvastatin, it is known that statin use can potentially cause drug-induced liver damage, including jaundice (Bjornsson et al., 2012; Bjornsson, 2021). Therefore, it is reasonable to hypothesize that the combination of amlodipine and atorvastatin may lead to jaundice, but further clinical research is needed to establish a causal relationship.

This study revealed that the combination of aspirin with ticagrelor or clopidogrel could potentially lead to bleeding in various organs. The Micromedex website also indicates that the combination of aspirin with ticagrelor or clopidogrel can cause bleeding as an adverse drug reaction (Merative. 2024). Furthermore, information from drug websites suggests that aspirin may reduce the effectiveness of ticagrelor in preventing blood clots, and the combination of aspirin with clopidogrel can result in gastrointestinal bleeding. A randomized controlled study conducted in 187 countries, involving 9,006 patients across 11 sites, demonstrated that the combination of aspirin with ticagrelor significantly increased the risk of bleeding compared to aspirin alone (Mehran et al., 2019). Another study showed that Ticagrelor combined with aspirin increased the risk of bleeding compared with ticagrelor alone (Dangas et al., 2020).

Peng’s study involving 47 patients with acute myocardial infarction demonstrated that the combination of aspirin with ticagrelor resulted in systemic bleeding (Peng and Li, 2022). Similarly, Xu’s study revealed that patients receiving ticagrelor or clopidogrel in combination were at a higher risk of major bleeding compared to those receiving aspirin alone (Xu et al., 2022). However, it is worth noting that there are also studies indicating that the combination of ticagrelor and aspirin does not increase the incidence of bleeding when compared to ticagrelor monotherapy. The variations in these findings may be attributed to differences in experimental design, patient characteristics, and other factors.

A study conducted by Arun Kalyanasundaram demonstrated that Clopidogrel alone can lead to major bleeding. However, when aspirin is combined with Clopidogrel, the risk of major bleeding increases. This finding is consistent with other studies that have reached the same conclusion (Hahn et al., 2019; Kalyanasundaram and Lincoff, 2011; Mehran et al., 2019; Watanabe et al., 2019). Therefore, the combination of aspirin with ticagrelor or clopidogrel does increase the occurrence of bleeding.

The findings of this study revealed that the combination of aspirin and nifedipine led to the occurrence of skin adverse reactions. However, no reports of adverse skin reactions caused by the interaction between aspirin and nifedipine were found on Micromedex and other drug websites (Merative. 2024). Nonetheless, there have been reported cases where patients who were being treated for Raynaud’s phenomenon with aspirin, nifedipine, colchicine, and naproxen developed severe skin reactions after 2 weeks (Aiempanakit et al., 2018),as well as hypersensitivity reactions to aspirin use (Banerji et al., 2023). Currently, no other studies have reported skin reactions caused by the combination of aspirin and nifedipine. Calcium channel blockers, including nifedipine, have been associated with inducing skin reactions, and the patients in this study were also taking aspirin (Davis et al., 2019). Additionally, Efendipine, another calcium channel blocker, has been reported by the World Health Organization to cause skin reactions (World Health Organization, 2018). Aspirin and nifedipine are commonly used in combination for the treatment of concurrent diseases; however, there is a lack of clinical trials evaluating the safety of this combination, and further research is needed by scholars in this area.

The results of this study revealed that the combination of vitamin B6 and vitamin C could potentially lead to the occurrence of chills in patients. However, there were no relevant reports found on Micromedex and other drug websites (Merative. 2024), and existing studies have provided limited safety reports on the two drugs. Existing studies have primarily focused on the beneficial effects of the combination of vitamin B6 and vitamin C, such as improving vascular risk and the biological status of pregnant women and newborns (Wang et al., 2015), as well as the effects of vitamins and minerals on cognitive health in middle-aged and elderly individuals (Rutjes et al., 2018). Additionally, there have been studies on the effects of other vitamins on overall health (Hwang and Song, 2020). However, there is a lack of studies specifically evaluating the safety of vitamins, both individually and in combination with other vitamins. Therefore, further research is needed to supplement the existing knowledge on the safety of vitamins alone and in combination with other vitamins.

Signal mining was performed separately in male and female patients, and the combination of amlodipine and atorvastatin for jaundice remained the strongest signal in both sexes. Second, the combination of aspirin and clopidogrel or ticagrelor has been found to cause a series of bleeding reactions, which have been shown to increase the probability of bleeding compared with aspirin or ticagrelo alone (Kalyanasundaram and Lincoff, 2011; Mehran et al., 2019). Vitamin B6 in combination with vitamin C was found to cause chills in female patients, while this signal was not found in male patients.

Signal mining is performed separately for two age groups: individuals aged 60 and above, and those below 60. The analysis includes signals related to jaundice resulting from the combined usage of amlodipine and atorvastatin, as well as signals associated with bleeding reactions caused by the concurrent use of aspirin with either clopidogrel or ticagrelor. Moreover, in the older age group (60 years and above), the occurrence of chills due to the combination of vitamin B6 and vitamin C is also investigated.

In patients under the age of 60, signals of palpitation associated with the combined use of irbesartan and felodipine have been identified. According to the drug website, the combination of felodipine and irbesartan can lead to elevated levels of blood potassium, which may result in irregular heartbeats. Irbesartan is an angiotensin II receptor antagonist that widens blood vessels and lowers blood pressure by blocking the angiotensin II receptor. Conversely, felodipine is a calcium channel blocker that reduces blood pressure by inhibiting the entry of calcium ions into cardiac muscle cells, thereby decreasing the contractility and heart rate. The combination of angiotensin II receptor blockers and calcium channel blockers has been shown to enhance the effectiveness of blood pressure reduction, thus improving the management of hypertension (Abraham et al., 2015; Lauder et al., 2023). Studies have indicated that palpitation is one of the adverse reactions associated with calcium channel antagonists, including felodipine (Cicardi et al., 2004; Elliott and Ram, 2011; Lorimer and Pringle, 1990; Meyers and Siu, 2011; Obermann et al., 2021; Salama, 2022; Wang et al., 2023). Furthermore, angiotensin II receptor inhibitors have been linked to cardiovascular diseases, with multiple animal studies demonstrating that they can cause heart enlargement even in the absence of elevated blood pressure (Agostini et al., 2024; Brunner, 2001; Salama, 2022). Other studies have indicated that the combination of angiotensin II receptor blockers and calcium channel blockers can also lead to arrhythmia and palpitations, underscoring the necessity for safety testing when using these two medications together. However, there is a lack of studies confirming whether the combination of these two drugs increases the incidence of palpitations in patients (Chi et al., 2016; Pongpanich et al., 2018). Moreover, it remains unclear whether the combination affects the pharmacokinetics of either drug *in vivo* or whether the palpitations result from one drug antagonizing or promoting the other, necessitating further clinical research.

Erythematosis has been observed as a result of the combination of compound Danshen tablets and nifedipine. No relevant reports were found on Micromedex and drug websitesregarding this specific combination (Merative, 2024). However, studies have indicated that nifedipine, used for treating high blood pressure, can cause various skin adverse reactions, including maculopapules (Knowles et al., 1998; Stern and Khalsa, 1989),photosensitivity reactions (Collins and Ferguson, 1993; Seggev and Lagstein, 1996)、drug-induced lupus erythematosus (He and Sawalha, 2018; Ranugha and Betkerur, 2018; Tuchinda et al., 2014) and other skin-related adverse drug reactions (Elliott and Ram, 2011; Lo et al., 2021). Compound Danshen tablets, as a Chinese patent medicine, primarily consist of salviorrhiza, Notoginseng, astragalus, borneol, and other components. These ingredients are commonly used in the treatment of cardiovascular and cerebrovascular diseases, promoting blood circulation, removing blood stasis, and improving microcirculation. Clinical studies have reported adverse reactions associated with compound Danshen tablets, such as stomach discomfort, heartburn, abnormal liver function, oral ulcers, and urinary albumin positivity (Li et al., 2020; Song et al., 2024; Yu et al., 2014; Zhang et al., 2023). However, limited research is available on the safety of compound Danshen tablets in combination with other medications. The erythematosis resulting from the combined use of these two drugs may be attributed to the interaction between the blood-activating and stasis-removing components of compound Danshen tablets and the vasodilation effects of nifedipine, leading to localized skin vasodilation. Nevertheless, this hypothesis has not been confirmed by studies, and there is a scarcity of research on adverse drug reactions related to the combined use of these medications. Furthermore, studies specifically investigating the combination of Chinese patent medicines with other drugs are lacking, therefore requiring further investigation by scholars in this field.

This study conducted signal mining on individuals of various genders and ages. The organs affected by bleeding caused by aspirin and other drugs differ between sexes. Females taking aspirin and other drugs are more susceptible to gastrointestinal bleeding, while males are prone to symptoms such as gastrointestinal bleeding, cerebral hemorrhage, hematochezia, hemoptysis, and other symptoms. Additionally, a signal of chills resulting from the combination of vitamin B6 and vitamin C was observed in women and elderly patients. Regrettably, there is a lack of clinical drug trials examining the combination of these drugs in various populations, necessitating the verification of the observed signal. In future studies, drug clinical trials can be conducted between different populations to speed up the process of personalized medicine.

### 4.1 Limitation

This study utilized a medical record report database, making it impossible to determine the exact number of patients using the related drugs and calculate the incidence or prevalence rates.

It is important to note that the research database used in this study relies on self-reporting, which introduces various reporting biases that may impact the validity and accuracy of the findings.

It is particularly important to note that our findings should only be used as reference information and do not replace the professional opinions of physicians, clinical pharmacists and other health professionals.

## Data Availability

The data analyzed in this study is subject to the following licenses/restrictions: The data that support the findings of this study are available from Adverse Drug Reaction Monitoring Center of Hubei Province but restrictions apply to the availability of these data, which were used under license for the current study, and so are not publicly available. Requests to access these datasets should be directed to Adverse Drug Reaction Monitoring Center of Hubei Province.
